# Quality and Publication of Emergency Medicine Trials Registered in ClinicalTrials.gov

**DOI:** 10.5811/westjem.2019.12.44096

**Published:** 2020-02-24

**Authors:** Lisa Calvocoressi, Jesse Reynolds, Benjamin Johnson, Meghan M. Warzoha, Megan Carroll, Federico E. Vaca, Lori Post, James Dziura

**Affiliations:** * Yale Center for Analytical Sciences, Yale School of Public Health, New Haven, Connecticut; † Yale School of Medicine, Department of Emergency Medicine, New Haven, Connecticut; ‡ Johns Hopkins Bloomberg School of Public Health, Department of Epidemiology, Baltimore, Maryland; § Northwestern University, Feinberg School of Medicine, Department of Emergency Medicine, Chicago, Illinois

## Abstract

**Introduction:**

Promoting emergency medicine (EM) clinical trials research remains a priority. To characterize the status of clinical EM research, this study assessed trial quality, funding source, and publication of EM clinical trials and compared EM and non-EM trials on these key metrics. We also examined the volume of EM trials and their subspecialty areas.

**Methods:**

We abstracted data from ClinicalTrials.gov (February 2000 – September 2013) and used individual study National Clinical Trial numbers to identify published trials (January 2007 – September 2016). We used descriptive statistics and chi-square tests to examine study characteristics by EM and non-EM status, and Kaplan-Meier curves and log-rank tests to compare time to publication of completed EM and non-EM studies.

**Results:**

We found 638 interventional EM trials and 59,512 non-EM interventional trials conducted in the United States between February 2000 and September 2013, registered on ClinicalTrials.gov. EM studies were significantly less likely than non-EM studies to be National Institutes of Health-funded or to evaluate a drug or biologic. However, EM studies had significantly larger sample sizes, and were significantly more likely to use randomization and blinding. Overall, 34.3% of EM and 26.0% of non-EM studies were published in peer-reviewed journals. By subspecialty, more EM trials concerned medical/surgical and psychiatric/neurological conditions than trauma.

**Conclusion:**

Although EM studies were less likely to have received federal or industry funding, and the EM portfolio consisted of only 638 trials over the 14-year study period, the quality of EM trials surpassed that of non-EM trials, based on indices such as randomization and blinding. This novel finding bodes well for the future of clinical EM research, as does the higher proportion of published EM than non-EM trials. Our study also revealed that trauma studies were under-represented among EM studies. Periodic assessment of EM trials with the metrics used here could provide an informative and valuable longitudinal view of progress in clinical EM research.

## INTRODUCTION

Over a decade ago, the Institute of Medicine (IOM) released three reports on the future of emergency medicine (EM) in the United States (US).^1^–^3^ Those reports called for: 1) enhancing EM and trauma care research through additional federal funding; 2) assessing research needs, gaps, and opportunities; and 3) encouraging academic medical centers to provide research time and facilities.^4^–^5^ Those recommendations prompted roundtable discussions focused on advancing research in three broad areas: trauma; neurological and psychiatric emergencies; and surgical or medical emergencies.^6^

In light of these reports and recommendations, we aimed to characterize the status of clinical EM research and to compare EM with non-EM studies. We restricted our assessment to clinical trials, essential components of evidence-based medicine and of interest to the National Institutes of Health (NIH), which promotes funding opportunities for high-quality, multisite EM trials.^7^ We based our assessment on several metrics: study quality; funding source; and dissemination of study findings. These metrics provide quantifiable measures of clinical EM research that can be used going forward to evaluate the characteristics and productivity of this research over time. In addition, we examined the distribution of trials across EM subspecialties (neurological/psychiatric, trauma, medical-surgical), which may help to direct funding to under-researched areas, and we examined the volume of EM registered trials by year and funding source, to assess trends.

We identified trials through ClinicalTrials.gov,^8^ which is the largest online trial registration and results reporting repository in the world and includes studies across medical disciplines.^9^ As trial registration is required at the time of study enrollment, ClinicalTrials.gov includes published and non-published studies, thereby providing a comprehensive listing of initiated trials. We used this information to compare EM and non-EM studies on the metrics of interest identified above, and to conduct EM-specific analyses on subspecialty and trends in registration. For dissemination of study findings, we examined whether the EM and non-EM trials registered in ClinicalTrials.gov were published in peer-reviewed journals.

## METHODS

### Data Abstraction

We abstracted data from the publicly available Aggregate Analysis of ClinicalTrials.gov (AACT) database, a relational database with multiple tables of downloadable data, provided by the Clinical Trials Transformation Initiative, to facilitate analysis of trial data registered in ClinicalTrials.gov.^10^ We restricted our search to studies conducted in the US and downloaded data on trials registered from the inception of the ClinicalTrials.gov website (February 2000) until September 2013.

### Search Strategy for Eligible EM and non-EM Trials in ClinicalTrials.gov

To identify potential EM studies, we first conducted an automated keyword search of the title and brief and detailed description fields of each study’s ClinicalTrials.gov record that was included in the AACT database We searched for the following terms: 1) emergency; 2) ER; 3) ED; 4) EM; and 5) acute care. Then, ClinicalTrial.gov administrators at our institution with expertise in protocol review and registration (JR, JD, AO, MW) manually reviewed these studies to identify which were truly EM studies, defined as studies that took place in the EM setting or studies that addressed medical issues related to EM. Initially, these administrators examined a subset of studies and collectively discussed and resolved any classification issues. Remaining studies were evaluated by one of these individuals using standardized criteria.

**Population Health Research Capsule**

What do we already know about this issue?

Efforts to advance rigorous emergency medicine (EM) clinical trials continue, but little is known about the current quality and characteristics of this research.

What was the research question?

Using ClinicalTrials.gov and PubMed data, how do EM and non-EM trials compare on funding, quality and publication of results?

What was the major finding of the study?

EM studies had less federal/industry funding, but their quality and likelihood of publication surpassed non-EM trials.

How does this improve population health?

We identified key metrics for monitoring and improving EM research. This paves the way for strengtheningthe EM evidence base and enhancing patient care.

Once the initial set of EM studies was identified, we used a machine learning algorithm based on the study title and brief and detailed description fields to build a text classifier that categorized EM and non-EM studies automatically. For studies identified as non-EM based on the keyword search above, we conducted a keyword search for “trauma” to search for additional EM studies. We applied the text classifier separately to studies that did and did not match on “trauma.” For each study, the text classifier generated an estimated probability of being an EM study. For each group based on keyword “trauma,” we sorted the studies by estimated probability, and stopped the searches after observing no EM studies below an estimated probability cutoff significantly lower than 1%. We then manually reviewed the studies with the highest EM study probability to determine whether any met criteria for inclusion as EM studies. We further narrowed our inclusion criteria for identified EM and non-EM studies to interventional trials, using the “study design” field in the AACT database to exclude observational studies.

### Variables

We included the following measures of study characteristics and quality that we extracted from the AACT database: 1) study phase; 2) intervention type; 3) sample size; 4) randomization; and 5) blinding. Following the methods described by Goswami and colleagues,^11^ we derived funding source from AACT database variables: 1) “agency” (NIH, US federal, industry, other); and 2) “sponsor type” (lead sponsor and collaborators). We categorized funding, based on the sponsor, as the following: 1) industry; 2) NIH and US federal funding; and 3) “other.”

We manually categorized EM interventional studies by research topic using the NIH Task Force on Research in the Emergency Setting criteria.^6^ We used AACT database fields “official title,” and the study’s “detailed description” to designate the research topic. Initially, two raters (JR and MW) categorized each study. Across 17 studies, chance-adjusted agreement was қ = .79. The original raters then trained one additional rater (AO) and one of these raters then examined each of the remaining studies. Raters could include a study under more than one substantive area, if appropriate.

### Identifying Published Studies

We turned to the published literature to assess dissemination because federal law requires that only a subset of registered trials report results in ClinicalTrials.gov, ie, applicable clinical trials that include investigations of a drug or biologic other than phase 1; investigations of Food and Drug Administration-regulated devices other than small feasibility studies and some studies of prototype devices; and pediatric post-market surveillance of devices.^12^

In July 2005, the National Library of Medicine began including the NCT number in the MEDLINE record when it was included in the published paper.^13^ Also in 2005, the International Committee of Medical Journal Editors began requiring trial registration as a condition of publication.^14^ Hence, we assumed a more complete listing of registered trials beginning in 2005. We began our search for publications with trials completed in January 2007 as we assumed that trials registered in 2005 would take at least two years to complete. We based this assumption on a review of a subset of studies registered in ClinicalTrials.gov by our institution where the average time from registration to study completion was 2.9 years. We included all studies completed up to the final date of data abstraction, September 27, 2013. We followed these completed studies through September 30, 2016, thus providing a minimum follow-up of three years to determine whether the study had been published.

To identify completed studies, we used the “study completion date” provided in ClinicalTrials.gov; if the study was missing that date but had a “primary completion date,” we used the latter. We used the same criteria to identify EM and non-EM published studies, ie, matching the unique ClinicalTrials.gov National Clinical Trial (NCT) identifier to the study’s MEDLINE record in PubMed.

### Statistical Analyses

To compare EM and non-EM studies on study characteristics and quality, funding source, and publications, we used descriptive statistics and chi-square tests. We compared time to publication for EM and non-EM studies using Kaplan-Meier curves and log-rank tests. We also used Kaplan-Meier methods to compare differences in time to publication for EM and non-EM studies stratified by funding source and study phase. We considered unpublished studies censored at the final date of potential publication, September 30, 2016. We conducted two EM-specific analyses: trends in EM trial registration by funding source and year (2000–2013), and proportions of EM trials in three sub-specialties (trauma, neurological and psychiatric emergencies, and surgical or medical emergencies). We used frequency distributions for these analyses.

We used SAS software, version 9.3 of the SAS System for Windows, copyright 2002–2012 (SAS Institute Inc., Cary, NC) for the initial search for EM and non-EM studies, for descriptive statistics, and for comparisons between EM and non-EM studies on trial characteristics. We used R: A Language and Environment for Statistical Computing, version 3.3.0, (R Foundation for Statistical Computing, Vienna, Austria) to develop the automatic text classifier, and to determine time to publication.

## RESULTS

### Identification of Emergency Medicine Trials

**Figure 1:**
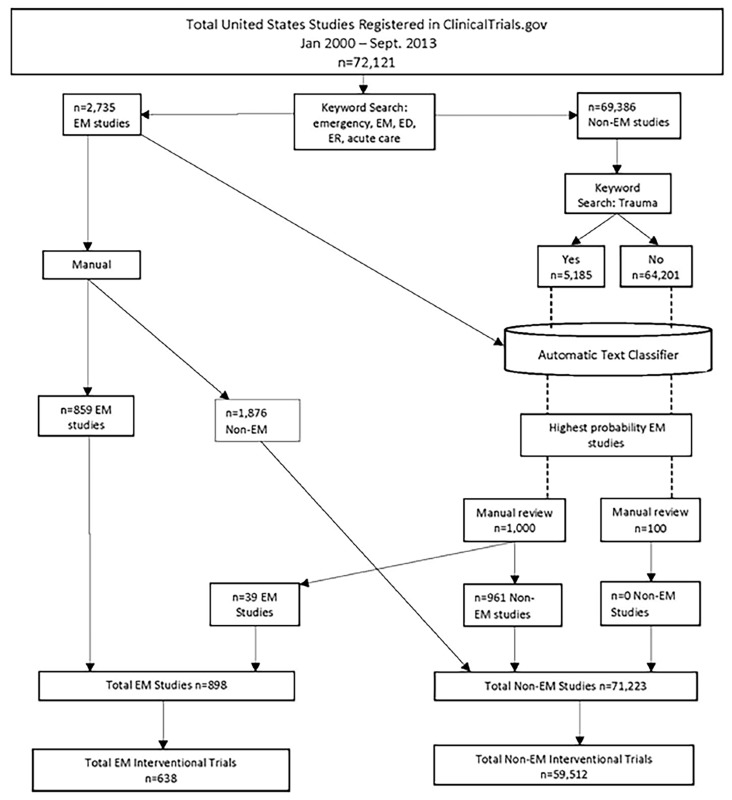
Emergency medicine (EM) and non-EM clinical trials inclusion flow chart. *ED*, emergency department; *ER*, emergency room.

As shown in Figure 1, there were over 72,000 US studies registered between January 2000 and September 2013. The first keyword search on emergency terms yielded 2,735 studies, which we used to develop the automatic text classifier. Using the text classifier and manual review as shown in the figure, we identified a total of 898 EM studies. Omitting observational studies, we found 638 interventional EM trials conducted in US-registered in ClinicalTrials.gov to September 27, 2013 and we identified 59,512 non-EM interventional trials conducted in the US during the same period.

### Characteristics and Quality of Registered Trials

Table 1 shows significant differences between EM and non-EM trials on study characteristics and indices of trial quality. More non-EM than EM trials evaluated a drug/biologic (74.2% vs 46.1%). EM trials were more likely to evaluate procedural, device, or behavioral interventions. Only about half of all EM trials reported any study phase, compared with three quarters of non-EM trials and fewer EM (45.6%) than non-EM studies (51.3%) were phase 2 or higher; thus, more non-EM trials met criteria for applicable clinical trials that required study results reporting. With respect to trial quality, EM studies were more likely to be randomized, to use blinding, and to have larger sample sizes. Among EM studies, 59% were classified as medical/surgical trials, 36% as neurological/psychiatric, and 17% as trials involving trauma patients. These proportions sum to greater than 100% and indicate that some trials investigated more than one substantive area.

### Funding

**Figure 2:**
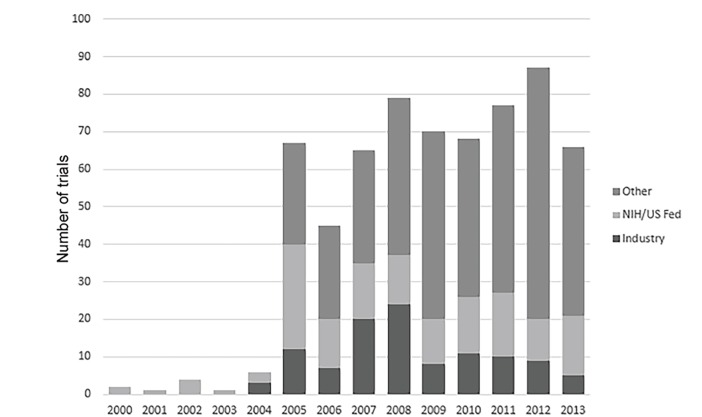
Number of trials registered in ClinicalTrials.gov by funding source from February 2000-September 2013. *NIH*, National Institutes of Health; *US Fed*, United States federal.

Table 1 also shows significant differences between EM and non-EM trials on funding source. Whereas 32.3% and 41.4% of non-EM trials were funded by the NIH or by industry, respectively; only 23.7% and 17.1% of EM trials were funded by these sources. Figure 2 displays registered EM clinical trials by year and funding. A marked increase in the number of registered trials occurred in 2005, the first year investigators registered trials that were funded by sources other than industry or government. Between 2007 and 2013 EM trial registration was fairly stable, with 65–87 newly registered trials per year. Throughout that period, EM studies continued to be dominated by “other” sources of funding.

### Publication of Trial Results

Between September 27, 2007, and September 30, 2016, 216 of 638 EM registered trials were completed (Table 2). Based on linking trial registration number with the PubMed record, we found that 74 (34.3%) of those studies were published in peer-reviewed journals three or more years after completion. Among non-EM studies, 5788 (26.0%) were published during that time frame. Of all completed studies, 97.7% of EM and 92.7% of non-EM studies had a study completion date in ClinicalTrials.gov. Among those, the proportions of EM and non-EM studies that were published within one year were 6.6% and 4.4%, respectively. At two years, 18.0% of EM and 11.9% of non-EM studies had been published in peer-reviewed journals.

**Figure 3:**
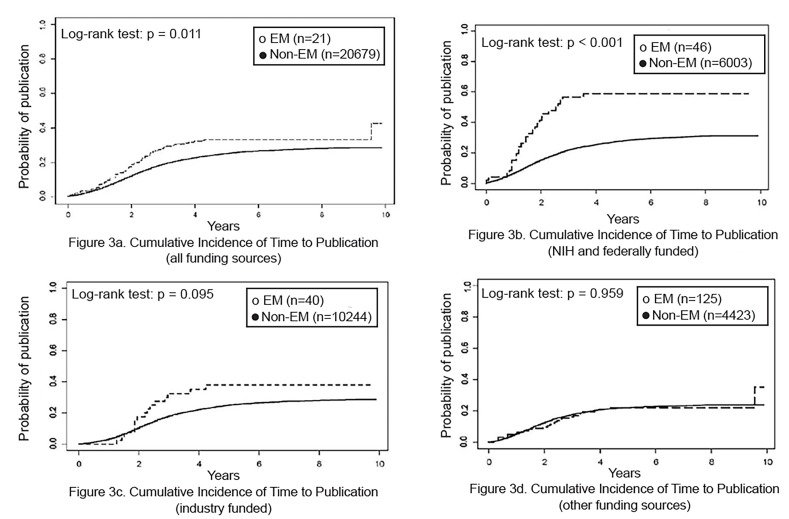
Cumulative incidence for time to publication overall and by funding source, emergency medicine (EM) and non-EM trials. *NIH*, National Institutes of Health.

Overall, EM studies were more likely than non-EM studies to be published (p = 0.011; Figure 3a). By funding source, NIH-funded EM studies were more likely than NIH-funded non-EM studies to be published (p<0.01; figure 3b). However, among industry- and other-funded studies, publication of EM and non-EM studies did not differ (Figures 3c–3d). In addition, publication of EM and non-EM studies did not differ by study phase (data not shown).

## DISCUSSION

Among US clinical trials registered in ClinicalTrials.gov, we found significant differences between EM and non-EM on trial characteristics and quality measures, funding sources, and dissemination of results through publication. Regarding trial characteristics, fewer EM than non-EM trials identified a study phase. This is consistent with our finding that fewer EM than non-EM trials assessed drug and biologic interventions; trials without phases typically assess behavioral interventions or devices. On measures of trial quality, EM compared favorably with non-EM trials; they were more likely to be randomized, to employ single or double blinding, and to include larger sample sizes. However, although of significantly higher quality, fewer EM than non-EM trials in our study received funding from industry or NIH and other federal agencies. There is broad consensus, dating back to the 2006 IOM reports, that there is a need to increase the conduct of EM clinical trials to expand the overall EM evidence base,^4^ and that increased funding is needed to meet that goal.^6^, ^15^ The good quality of EM studies that we observed suggests that EM is well positioned to increase its base of funded studies. Indeed, the number of EM projects submitted to NIH has increased in recent years.^17^ Still, the number of NIH-funded projects remains low compared with other medical specialties.^16^ For example, EM-funded applications comprised less than 1% of the 2014 NIH research budget, paling in comparison to funding for sleep disorders and rehabilitation research.^16^,^17^

Continuing to develop, expand, and promote the pursuit of broad and diverse research within EM may help to boost federal funding.^18^,^19^ In that regard, we found that subspecialty areas were not equally represented among EM trials. Nearly 60% of these trials examined medical and surgical topics; only 17% studied trauma. Similarly, Roberts and colleagues (2005) reported that few clinical trials are conducted on traumatic injuries compared with trials across a range of chronic and infectious disease conditions. The authors added that “funding for trauma research is less than for almost any other cause of human suffering” (p. 1095).^20^ There are formidable challenges to conducting research in the EM setting, including the fast-paced/high-pressure environment, time constraints, and difficulties obtaining consent.^21^ In research involving trauma patients, these challenges may be amplified and contribute to the paucity of studies in this area. Increasing and prioritizing funding, as well as addressing ethical issues, expanding and creating trauma research networks, and developing a standard template for trauma research are some suggestions that emerged from the NIH Roundtable on Emergency Trauma Research convened to enhance research in this field.^22^

Of utmost importance is the dissemination of research findings. Ross and colleagues point out that when trial results are not disseminated, scientific knowledge suffers though potential redundancy of studies and inaccuracies about clinical evidence; commitment to trial participants is violated; and the investigator’s ethical obligation to disseminate findings of studies is unmet.^23^ In our study, overall, EM trials were more likely than non-EM to be published at all measured time points. Still, we found that only 34.1% and 26.1%, of EM and non-EM trials completed between 2007 and 2013 were published, respectively, when allotting at least three years from study completion to publication. These findings are largely consistent with those of Huser and Cimino who found that 27.8% of their sample of phase 2 or higher studies registered in ClinicalTrials.gov had been published within three years, when they searched for publications via a unique identifier (eg, NCT number or PubMed ID).^9^

Huser and Cimino note that their search strategy, similar to our method, tends to yield a lower proportion of published works than manual article-retrieval methods. Based on studies they reviewed, these investigators report that 46%–68% of registered trials had published results when searches were conducted manually. Given the volume of studies we examined, it was not feasible to conduct a comprehensive manual search and we recognize that our findings likely underestimate the proportions of registered trials that were published. However, our method of identifying publications was consistent across EM and non-EM studies and should thus have obtained an unbiased comparison between these groups. Indeed, our decision to examine dissemination of results based on published papers likely avoided bias that could have arisen had we relied on results reported in the ClinicalTrials.gov database. More non-EM than EM trials were phase 2 or higher and investigated a drug or biologic; thus, more non-EM than EM trials would have met the definition of an applicable clinical trial that requires results reporting in ClinicalTrials.gov.

## LIMITATIONS

We limited our study to trials conducted in the US and these results may not generalize to studies of trials conducted in other countries. In addition, we may have missed some relevant trials. First, some trials may have gone unregistered, particularly from the inception of the ClinicalTrials.gov registry until 2005 when the International Committee of Medical Journal Editors (ICMJE) began requiring registration for publication in one of its journals.^14^ Second, ICMJE-mandated registration gives investigators the option of several online registries (eg, World Health Organization International Clinical Trial Registry Platform or ClinicalTrials.gov); by limiting our search to ClinicalTrials.gov, we would have overlooked studies in those registries as well.

Although ICMJE requires investigators to register all trials conducted on human subjects, federal law requires registration only of studies that are applicable clinical trials (ACTs).^12^ We found that EM trials were less likely than non-EM to be ACTs, so more EM than non-EM trials may have gone unregistered. In addition, by identifying registered EM trials through search words “emergency,” “ER,” “ED,” “EM,” “acute care,” and “trauma,” we may have overlooked and misclassified some EM trials. Furthermore, we did not formally examine the validity of the automatic text classifier that we used to identify EM studies, but we did conduct manual reviews of a subset of the articles that the text classifier prioritized.

With respect to classifying EM studies by subspecialty, we did examine agreement between our initial two raters of EM, but we did not formally compare agreement with a third rater subsequently trained by the initial raters. Our use of NCT number to identify published studies likely underestimated the proportion of published trials; studies that conduct manual searches of the PubMed database find a larger proportion of published studies. 9 Moreover, we restricted our search for publications to MEDLINE, potentially missing published works in other online databases such as the Cochrane Library, Cumulative Index to Nursing and Allied Health Literature, or Excerpta Medica Database.

We collected data on studies registered through September 27, 2013, and concluded our follow-up of published studies in September 2016. It is possible that characteristics of more recently registered trials may differ and our data may not demonstrate newer trends in research. Future studies should examine more recent data and might also extend the follow-up for published work. Future studies should also endeavor to assess clinical EM research and its progress in relation to other specific medical specialties and subspecialties. We compared EM studies with non-EM, the latter being a very broad comparator, likely with wide-ranging differences in study characteristics and quality and publication status by medical specialty.

## CONCLUSION

Given the commitment to expand and advance clinical EM research, periodic assessment using quality indicators can provide informative quantitative data to assess its progress. This study used several key metrics for evaluating EM clinical trials including trial quality, funding source, and dissemination of study findings. Data for studies completed through September 2013 and followed for publication through September 2016 indicated that the EM portfolio consisted of only 638 trials over the 14-year study period and that trauma research accounted for only a small proportion of EM studies. Further, compared with non-EM studies, EM studies were less likely to have received federal or industry funding. Nonetheless, the quality of EM trials surpassed that of non-EM trials, based on indices such as randomization and blinding. This novel finding bodes well for the future and advancement of EM research, as does the higher proportion of published EM vs non-EM trials. Periodic assessment of EM trials with the metrics used here will help to provide a valuable longitudinal view of progress in clinical EM research.

## Figures and Tables

**Table 1:** Characteristics of emergency medicine and non-emergency medicine intervention studies.

Study characteristic	Emergency medicine trials (n = 638) Number (%)	Non-emergency medicine trials (n = 59,512) Number (%)	P-value
Funding source			<0.001
National Institutes of Health/US federal	151 (23.7)	19,197 (32.3)	
Industry	109 (17.1)	24,309 (41.4)	
Other	378 (59.3)	16,006 (26.9)	
Phase			<0.001
N/A	310 (48.6)	14,614 (24.6)	
Phase 0–1	37 (5.8)	14,337 (24.0)	
Phase 2	106 (16.6)	17,052 (28.6)	
Phase 3	79 (12.4)	8,454 (14.2)	
Phase 4	106 (16.6)	5,055 (8.5)	
Intervention			
Drug/Biologic	294 (46.1)	44,153 (74.2)	<0.001
Procedure	84 (12.7)	5,891 (9.0)	0.01
Device	75 (11.8)	4,581 (7.7)	<0.001
Behavioral	125 (19.6)	6,664 (11.2)	<0.001
Other	126 (19.8)	10,891 (18.3)	0.28
Randomized	534 (83.4)	34,105(57.3)	<0.001
Blinded			
Single	129 (20.2)	5,204 (8.7)	<0.001
Double	221 (34.6)	17,895 (30.1)	0.04
Sample size			<0.001
<50	96 (15.3)	24,693 (43.3)	
50–100	118 (21.5)	12,456 (21.9)	
>100	414 (63.2)	19,856 (34.8)	
Topic			
Neurological/Psychiatric	230 (36.1)	N/A	
Trauma	110 (17.2)	N/A	
Medical/Surgical	378 (59.3)	N/A	

*US*, United States; *N/A*, not available.

**Table 2:** Completed and published emergency medicine and non-emergency medicine intervention studies included in ClinicalTrials.gov registry, September 2007-September 2016.

Completed and published studies	Emergency medicine Number (%)	Non-emergency medicine Number (%)
All completed studies	216	22,298
Published in three or more years	74 (34.3)	5,788 (26.0)
Missing/incorrect study completion date	4	1,619
Completed studies with study completion date	211	20,679
Published in three or more years	72 (34.1)	5,390 (26.1)
Published within two years	38 (18.0)	2,459 (11.9)
Published within one year	14 (6.6)	903 (4.4)

## References

[1] Institute of Medicine Committee on the Future of Emergency Care in the United States Health System. Hospital-based emergency care: at the breaking point.

[2] Institute of Medicine Committee on the Future of Emergency Care in the United States Health System. Emergency care for children: growing pains.

[3] Institute of Medicine Committee on the Future of Emergency Care in the United States Health System. Emergency medical services at the crossroads.

[4] Handel DA, Sklar DP, Hollander JE (2007). Executive summary: the Institute of Medicine Report and the future of academic emergency medicine: the Society for Academic Emergency Medicine and Association of Academic Chairs in Emergency Medicine panel: Association of American Medical Colleges annual meeting, October 28, 2006. Acad Emerg Med.

[5] Institute of Medicine of the National Academies (2006). Fact Sheet. The future of emergency care: key findings and recommendations.

[6] National Institutes of Health Request for information: soliciting input on current needs in emergency medicine research.

[7] Department of Health and Human Services Part 1. Overview Information.

[8] ClinicalTrials.gov https://clinicaltrials.gov/.

[9] Huser V, Cimino JJ (2013). Linking ClinicalTrials.gov and PubMed to track results of interventional human clinical trials. PLoS One.

[10] Clinical Trials Transformation Initiative State of clinical trials. AACT database.

[11] Goswami ND, Pfeiffer CD, Horton JR (2013). The state of infectious diseases clinical trials: a systematic review of ClinicalTrials.gov. PLoS One.

[12] ClinicalTrials.gov FDAAA 801 and the Final Rule.

[13] U.S. National Library of Medicine Clinical Trial Registry Numbers in MEDLINE®/PubMed® Records.

[14] International Committee of Medical Journal Editors Clinical Trials Registration.

[15] Bounes V, Dehours E, Houze-Cerfon V (2013). Quality of publications in emergency medicine. Am J Emerg Med.

[16] Brown J (2016). National Institutes of Health support for clinical emergency care research 2011–2014. Ann Emerg Med.

[17] Baren JM, Cairns CB, Neumar RW (2016). National Institutes of Health funding of emergency care research: feast or famine?. Ann Emerg Med.

[18] Nocera R, Ramoska EA, Hamilton RJ (2016). Building a resident research program in emergency medicine. Intern Emerg Med.

[19] Ranney ML, Limkakeng AT, Carr B (2015). Improving the emergency care research investigator pipeline: SAEM/ACEP recommendations. Acad Emerg Med.

[20] Roberts I, Shakur H, Edwards P (2005). Trauma care research and the war on uncertainty. BMJ.

[21] El-Menyar A, Asim M, Latifi R (2016). Research in emergency and critical care settings: debates, obstacles and solutions. Sci Eng Ethics.

[22] Cairns CB, Maier RV, Adeoye O (2010). NIH roundtable on emergency trauma research. Ann Emerg Med.

[23] Ross JS, Tse T, Zarin DA (2012). Publication of NIH funded trials registered in ClinicalTrials.gov: cross sectional analysis. BMJ.

